# Use of Biosimilar Follicle-Stimulating Hormone in Asthenozoospermic Infertile Patients: A Multicentric Study

**DOI:** 10.3390/jcm9051478

**Published:** 2020-05-14

**Authors:** Maurizio De Rocco Ponce, Carlo Foresta, Rocco Rago, Alessandro Dal Lago, Giancarlo Balercia, Aldo Eugenio Calogero, Sandro La Vignera, Ilaria Cosci, Andrea Di Nisio, Andrea Garolla

**Affiliations:** 1 UOC Andrologia e Medicina della Riproduzione, Azienda Ospedaliera Università di Padova, Dipartimento di Medicina, 35126 Padova, Italy; carlo.foresta@unipd.it (C.F.); ilaria.cosci@yahoo.it (I.C.); andrea.dinisio@gmail.com (A.D.N.); 2 Unità di Fisiopatologia della Riproduzione e Andrologia, Ospedale Sandro Pertini, 00157 Roma, Italy; roccorago1@gmail.com (R.R.); alessandro.dallago@aslroma2.it (A.D.L.); 3 Endocrinologia, Dipartimento di Scienze Cliniche e Molecolari, Università Politecnica delle Marche, 60121 Ancona, Italy; g.balercia@univpm.it; 4 Dipartimento di Medicina Clinica e Sperimentale, Università di Catania, 95124 Catania, Italy; acaloger@unict.it (A.E.C.); sandrolavignera@unict.it (S.L.V.)

**Keywords:** male infertility, follicle-stimulating hormone, biosimilar FSH, asthenozoospermia, sperm DNA fragmentation

## Abstract

There is increasing data in favour of follicle-stimulating hormone (FSH) therapy in patients with oligo-asthenozoospermia and normal-range gonadotropins in order to increase sperm count and above all sperm motility. Some studies showed an improvement in DNA fragmentation and spontaneous pregnancy. Recently, biosimilar FSH has been marketed with the same indications. We performed a retrospective multicentric case-control study involving 147 asthenozoospermic patients between 18 and 45 years of age. A total of 97 patients were treated with biosimilar FSH 150 UI three times a week for 3 months, while 50 control subjects received no treatment. Patients were evaluated at baseline and after 3 months with semen analysis including DNA fragmentation, testicular colour Doppler ultrasound, and blood tests. Spontaneous pregnancies were recorded during a further follow-up period of 6 months. Treated patients showed after treatment a statistically significant increase in sperm concentration, total sperm count, and total motile sperm, as well as improved progressive motility and non-progressive motility. DNA fragmentation showed a significant reduction. Conversely, in the control group, no significant change was found. Pregnancy rate was significantly higher in treated patients. These data suggest comparable efficacy of biosimilar FSH in the treatment of male infertility; however, larger studies are needed to confirm our results.

## 1. Introduction

The gonadotropin follicle-stimulating hormone (FSH) is a glycoprotein polypeptide synthesized and secreted by the gonadotropic cells of the anterior pituitary gland upon the pulsatile stimulus of the gonadotropin-releasing hormone (GnRH) produced by the hypothalamus [1]. After secretion, it reaches the gonads where, in the male, FSH has an important role in testicular development and spermatogenesis, as demonstrated by several studies in both animal models and humans [2,3,4,5,6,7]. FSH acts on testicular Sertoli cells, enhancing their trophic function for tubular spermatogenetic cells. Sertoli cells create a favourable environment in which spermatogonia proliferate and mature to spermatozoa [8,9]. The transduction of FSH signal to target tissues is mediated by a specific G protein-coupled receptor (FSHR), which in turn activates different signalling pathways and a number of intracellular effectors, resulting in the transcription of target genes [10,11]. Structurally, FSH consists of a heterodimer formed by an α and a β subunit; the α-subunit is common to both gonadotropins and thyrotropin, whereas the β-chain is responsible for their biological specificity [12]. Each FSH subunit can have two asparagine-linked carbohydrate side chains, and the anterior pituitary gland produces several FSH isoforms with heterogeneity of the carbohydrate side chains, which influence their molecular weight, isoelectric properties, bioactivity, immunoreactivity, and circulating half-life [13]. In particular, FSH is characterized by a microheterogeneity, resulting from oligosaccharide structure variation, and a macroheterogeneity, arising from partial FSHβ subunit glycosylation. An impact deriving from these carbohydrate variations on human FSH function has been suggested [14,15]. In facts, some differences in testicular gene expression responses to different FSH glycoforms have been demonstrated with in vivo animal experiments [16]. On the basis of its physiological capacity to stimulate spermatogenesis, FSH has been initially used with therapeutic purposes to treat hypogonadotropic hypogonadism (HH) and its associated male infertility [17]. In these cases, depending on the underlying cause of HH, a full restoration of normal seminal parameters may not be achieved [18]. However, the positive results obtained in this setting suggested the use of gonadotropins to stimulate spermatogenesis in patients with idiopathic oligozoospermia or oligo-asthenozoospermia and normal-range gonadotropins. The rationale derived also from studies in non-human primates demonstrating that hemicastration induced raised FSH levels and increased volume of the contralateral testis with enhanced spermatogonia proliferation [19]. Moreover, exogenous FSH administration significantly increases the number of spermatogonia in monkeys, suggesting that spermatogenesis can be overstimulated above the physiological rate [20,21]. Other data described in men after monolateral orchiectomy for testicular malignancy confirmed in humans the possibility of compensatory increase of spermatogenesis [22]. There is an increasing amount of data in favour of FSH therapy to improve sperm count and motility in men with idiopathic oligozoospermia or oligo-asthenozoospermia and normal-range gonadotropins [23]. On the contrary, high levels of gonadotrophins are recognized as a predictive factor for unresponsiveness in oligozoospermic patients [23,24,25,26]. It has been demonstrated that the presence of a maturation arrest in testicular fine-needle aspiration cytology is associated with FSH treatment failure [27]. Likewise, maturation arrest is associated to the presence of high spermatid count in semen, and this has been demonstrated to negatively predict the efficacy of FSH therapy [28]. Moreover, gene polymorphisms of FSH receptor can modify the response to FSH treatment, whereas other predictive factors have been suggested but are still to be fully defined [29]. Meta-analyses show that among conventional seminal parameters, motility is the one that improves the most [10]. Beyond that, some studies have shown an improvement in sperm structure and DNA fragmentation after FSH therapy, and promising results have been found particularly in patients with high levels of DNA fragmentation index (DFI) [30,31]. These findings may explain the improved results in oocyte fertilization and pregnancy rate sometimes obtained despite the absence of any improvement of classical seminal parameters in couples undergoing artificial reproductive therapy (ART) [32]. To the same extent, in infertile couples with idiopathic male factor, FSH (either human or recombinant) seems to be effective in increasing spontaneous pregnancy rates [33].

A protein-based product used for therapeutic purposes, just like FSH, is defined a “biological” product. The first generation of biological product was extracted from biological tissues. Successively, the development of recombinant DNA technology allowed for the obtaining of recombinant protein-based drugs that almost completely replace extractive proteins. FSH is nowadays produced by recombinant DNA technology (rFSH) or still purified from urine (hpFSH). After patent expiration of the original biological product, called “originator”, biosimilar products have been produced. A biosimilar product is a biological medicine obtained with recombinant DNA technology by other manufacturers different from the pharmaceutical industry producing the originator drug. Only minor differences in clinically inactive components are allowed in biosimilar products [34], and therefore they are supposed to retain the same efficacy and safety profile as the originator medicine and are approved for clinical use [35]. The use of biosimilar medicines may provide an improvement in cost-effectiveness of pharmacological treatments thanks to lower cost of these products compared to originator medicines [36].

The biosimilar alfa-follitropin (Bemfola, Finox AG, Burgdorf, Switzerland) is a biological medicine similar to the originator (Gonal-f, Merk Serono, Darmstadt, Germany). It is the first biosimilar FSH commercialized in Europe, and was approved by the European Medicines Agency (EMA) in 2014 [37]. As expected, biosimilar FSH present pharmacodynamic, pharmacokinetic, and toxicological profiles similar to the originator, and thereby it has been licensed for all indications of the reference product [38,39]. Clinical efficacy of biosimilar FSH has been demonstrated in a single-blind, randomized, parallel-group, multicentre study conducted in 15 European centres for women undergoing assisted reproduction techniques [40]. In another study comparing biochemical differences between originator and biosimilar FSH, the authors found some differences in the site-specific glycosylation at asparagine (Asn) 52 of the α-subunit of FSH, with possible implication in different capacity of receptor activation. Moreover, they reported a higher batch-to-batch variability of Bemfola compared to the originator Gonal-f [41]. More recently, Riccetti et al. performed a study investigating in vitro bioactivity and glycosylation patterns of two biosimilar FSH including Bemfola. The results confirmed distinct glycosylation profiles likely due to different source cell lines. Despite these peculiarities, no dissimilar preparation-specific intracellular signals or steroid synthesis in response to physiological concentrations were found. These data demonstrated similar bioactivity, and overall structural homogeneity of originator and biosimilar FSH [42].

In spite of data showing sperm motility as a sensitive parameter improving during FSH therapy, to our knowledge, there is no study that specifically examines the effects of biosimilar FSH therapy on sperm motility in asthenozoospermic infertile patients. The aim of this multicentric study was to evaluate the effect of biosimilar FSH on sperm motility in infertile patients affected by asthenozoospermia. Therefore, our primary objective was to compare progressive sperm motility and total motile sperm before and after treatment. Secondary objectives were to check the impact of the therapy on sperm DNA fragmentation and on spontaneous pregnancy rate.

## 2. Experimental Section

This is a retrospective multicentric case-control study involving four Italian andrological centres: (i) The Unit of Andrology and Reproductive Medicine—Padova University Hospital, (ii) the Unit of Pathophysiology of Reproduction and Andrology—Roma 2 University Hospital, (iii) the Unit of Andrology and Endocrinology—Catania University Hospital, and (iv) the Unit of Andrology and Metabolic Diseases—Ancona University Hospital. The study protocol was approved by the local ethics committee of Padova University Hospital (protocol number 2591P).

Cases were recruited among patients attending the outpatient clinics for fertility problems (i.e., inability to achieve a pregnancy despite a period of 12 months or more of regular unprotected intercourse). Inclusion criteria were age from 18 to 45 years old and infertility associated with confirmed asthenozoospermia according to the 2010 WHO criteria for semen analysis (progressive motility <32% or total motility <40%), independently from other sperm parameters. Exclusion criteria were FSH > 8 IU/L, seminal infections, anti-sperm antibodies >50%, abnormal karyotype or mutations of the CFTR gene, previous or actual oncologic problems, and severe renal or liver disease. We excluded couples with a female partner aged >35 years old, ovulatory disorders, tubal factor, uterine disease or endocrine abnormalities evaluated by hormone assessment, pelvic ultrasound examination, and hysterosalpingography. Control patients were recruited among subjects attending the outpatient clinics for fertility problems with the same inclusion and exclusion criteria as cases.

All methods were carried out in accordance with international guidelines and local protocols and regulations. At baseline (T0), all patients were evaluated by semen analysis according to 2010 WHO guidelines and criteria, testicular colour Doppler ultrasound, and blood tests for hormones (total testosterone, FSH and LH). Additionally, the patients recruited in Padova were also evaluated for sperm DNA fragmentation by TUNEL test. Both seminal analysis and TUNEL tests were performed via duplicate analysis by two different trained operators. Human semen samples were obtained by masturbation in sterile containers. Patients were instructed to collect the sample after 2–5 days of sexual abstinence. Semen samples were allowed to liquefy at a temperature of 37 °C for 30 min. Thereafter, they were examined according to 2010 WHO criteria to obtain main seminal parameters. Evaluation of the presence of sperm antibodies was carried out using the Sperm-Mar test (Ortho Diagnostic System, Milan, Italy). Part of the semen sample was used to perform a sperm culture. After semen analysis, we immediately evaluated DNA fragmentation. DNA fragmentation assessment was performed after washing in phosphate-buffered saline (PBS) and centrifuging at 1200× *g* for 5 min at room temperature. The pellets obtained were resuspended in PBS and samples analysed by TUNEL test. As reported by Sharma et al. [43], TUNEL test is a method to evaluate sperm DNA fragmentation. It is a sensitive test that detects both single-strand breaks and double-strand breaks. To perform TUNEL test, spermatozoa were fixed in paraformaldehyde (4%) at room temperature for 45 min, then washed with phosphate-buffered saline, and finally centrifuged at 1200× *g* for 5 min at room temperature. Pellets obtained were then resuspended and permeabilized with 200 μL of 0.1% Triton X-100 in 0.1% sodium citrate solution (Sigma-Aldrich, St. Louise, MO, USA) at −20 °C for 4 min. Thereafter, samples were washed with bovine serum albumin (BSA) solution 0.1% and then centrifuged at 1200× *g* for 5 min at room temperature. For the TUNEL assay, we used the Cell Death Detection Kit by Roche Diagnostics, Milan, Italy. According to the manufacturer’s instructions, to distinguish permeabilized spermatozoa, we used isothiocyanate–dUTP as label and propidium iodide as counterstaining (Sigma-Aldrich, St. Louise, MO, USA). For positive controls, we used cells previously incubated for 45 min with with DNAse I (1 μg/mL, Roche Diagnostics, Milan, Italy) at 37 °C. Negative controls were cells incubated with no enzyme. Cell analysis was finally carried out using a FACScan with Cellquest software (Becton Dickinson, Oxford, United Kingdom). Results are expressed with a percentage that represents the proportion of spermatozoa with fragmented DNA.

FSH treatment consisted of 150 UI three times a week for 3 months of biosimilar FSH (Bemfola 75 UI/0.125 mL; Finox AG, Burgdorf, Switzerland). After the 3 month treatment (T1), all patients were re-evaluated for the same variables as in T0. Spontaneous pregnancies were recorded during FSH treatment and during a further follow-up time of 6 months after the end of therapy (i.e., up to 9 months after the start of the study). Control patients were evaluated in the same way and performed the same analysis both at baseline and after a period of 3 months without any treatment. Moreover, spontaneous pregnancies were recorded for 9 months after the start of the study, the same as treated patients.

Statistical analysis was performed using SPSS 23.0 software for Windows (SPSS, Chicago, IL, USA). The results are expressed as means ± standard deviation (SD) for continuous variables, whereas categorical variables are expressed as percentage. To confirm normality of distribution, we used the Kolmogorov–Smirnov test, and variables without a normal distribution were log-transformed. Comparison between groups was carried out using unpaired Student’s *t*-test. For differences in continuous variables between T0 and T1, a repeated-measures ANOVA was performed. To test differences between time-points, a covariate analysis including age was performed as well as a post-hoc analysis with Bonferroni–Holm correction for multiple comparisons. Then, a Welch test was performed if homogeneity of variances assumption was violated. For categorical variables, differences between groups were compared using Pearson’s chi-squared test (or Fisher’s exact test for frequencies of 5 or less). *p*-values were considered statistically significant if <0.05.

## 3. Results

Ninety-seven infertile asthenozoospermic patients (cases) and 50 control patients were recruited from the four participant centres. Table 1 shows summarized clinical characteristics of patients and controls recorded at T0 (basal) and T1 (after 3 months of FSH treatment or follow-up, respectively, for cases and controls). It is worth nothing that there was no statistically significant difference between the two groups at T0 (all *p* > 0.05).

Treated patients showed a statistically significant increase in sperm concentration, total sperm count, and total motile sperm, as well as improved progressive motility and non-progressive motility. Immotile sperm percentage had a parallel significant decrease.

Along with seminal changes, testicular volume increased, whereas DNA fragmentation showed a significant reduction after 3 months of FSH therapy.

On the other hand, in the control group, no statistically significant difference was found at 3 months from baseline. In particular, this group had no change in total motile sperm count at T1. By repeated-measures ANOVA, we evaluated whether there was a significantly different effect of time (T0–T1) between the two groups (case–controls). The interaction time-by-group was significantly different for seminal volume, sperm total count, total motile sperm count, progressive and non-progressive motility, immotile sperm, normal morphology, FSH levels, right testicular volume, total testicular volume, and DNA fragmentation (Table 1).

**Figure 1:**
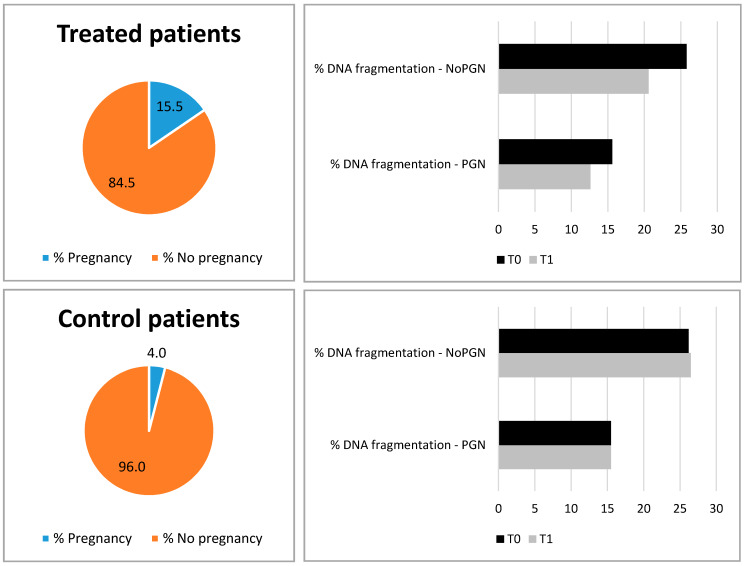
Pregnancy rate (% of couples at T1) of treated patients and control patients. DNA fragmentation observed (%) at T0 (black bars) and T1 (grey bars) in treated patients and control patient is reported, divided on the basis of positive or negative pregnancy outcome. PGN = pregnancy, NoPGN = no pregnancy.

Regarding fertility outcome, 15/97 treated patients (15.5%) had spontaneous pregnancy within 6 months from the end of therapy and 12/97 (12.3%) had full term delivery. Among control patients only 2/50 couples (4%) had spontaneous pregnancy and full-term delivery (Figure 1). The difference in pregnancy rate between cases and controls is statistically significant (*p* = 0.031).

We thereafter analysed the characteristics of couples who obtained pregnancy in comparison to those who did not. No difference was found in common seminal parameters, but the only predictive variable was the TUNEL test. In particular, DNA fragmentation was significantly lower in couples who obtained pregnancy compared to those that did not. This characteristic was observed both at baseline and at T1 and both in treated patients and control group (see Table 2 and Figure 1). Indeed, in repeated-measure ANOVA, the pregnancy outcome was significantly associated with reduced DNA fragmentation (*p* = 0.001).

## 4. Discussion

This study explored the effect of biosimilar FSH on seminal quality in asthenozoospermic patients. We carried out a retrospective case–control study investigating the effect of 3 months therapy on classical seminal parameters and DNA fragmentation. Moreover, we analysed the effect of treatment on spontaneous pregnancy rate observed at 6 months follow-up after the end of treatment. Treated patients and controls were recruited from the same andrological centres and following the same inclusion and exclusion criteria. At baseline, case and control patients did not have any statistical difference with regards to age, seminal parameters, DNA fragmentation, hormonal status, testicular volume, and partner’s age.

Treatment consisted of biosimilar FSH was administered at the dose of 150 UI 3 times a week for 3 months, a common scheme among previously published clinical trials on FSH in male idiopathic infertility [7].

Previous data have already demonstrated positive effects of recombinant or purified FSH on sperm concentration [27,30,44] and sperm motility [27,44], whereas previous data about sperm morphology are conflictive [23]. We confirmed a significantly increase of sperm concentration, sperm total count, progressive motility, non-progressive motility, and total motile sperm count in treated patients vs. controls. Moreover, our results on the percentage of normal morphology spermatozoa were significantly positive with FSH therapy, whereas no change was detected in untreated patients. The testicular volume also responded to biosimilar FSH with statistically increased size, probably as a consequence of enhanced tubular proliferation in accordance with previous animal studies [19]. In our study, sperm DNA fragmentation measured by TUNEL test decreased in treated patients similarly to the described data in men with idiopathic oligo-astheno-teratozoospermia [30].

In this study, pregnancies were recorded during 9 months from baseline both for treated and control patients. Treatment with biosimilar FSH was associated with statistically better pregnancy rate. However, the achievement of pregnancy was not associated with the improvement of seminal parameters supporting previous studies in which FSH therapy has been associated with increased pregnancy rate despite the absence of a clear correlation between sperm parameters and pregnancy rate [32]. A possible explanation may be the improvement of sperm DNA fragmentation, as shown by previous investigations in which a decrease of sperm DNA fragmentation obtained with recombinant FSH therapy was associated with better fertility outcomes [31]. Interestingly, among treated patients, those who achieved pregnancy also showed a greater relative improvement in TUNEL test in response to treatment compared to those who did not have a pregnancy, even though this trend was not statistically significant. Nevertheless, we cannot exclude the fact that a better spontaneous pregnancy rate in treated patients was due to a persistent improvement of seminal parameters after FSH treatment over the follow-up period.

We finally tried to investigate which characteristics at baseline could predict FSH response in terms of pregnancies. We found that patients who obtained a pregnancy did not differ statistically from those who did not conceive in any hormonal or classical seminal parameter, except for sperm DNA fragmentation. In contrast, sperm DNA fragmentation at baseline was much smaller in couples who obtained a pregnancy in both case and control couples.

The major limitations of this study were (1) the relatively small sample size, especially with regard to evaluation of pregnancy rate, and (2) the retrospective study design. We should also consider that other doses of FSH and other treatment lengths have been proposed in infertile men [7], and that different treatment regimens may induce different results [23]. Future research should investigate these aspects deeper, hopefully through using prospective randomized, double-blind, placebo-controlled studies and larger populations to ensure scientifically sound answers.

## 5. Conclusions

This is the first study showing significant improvement of sperm parameters induced by biosimilar FSH in asthenozoospermic infertile patients. In particular, important effects were observed on total motile sperm count and DNA fragmentation. The latter parameter was even related to better fertility outcomes in treated patients compared to controls. Further studies on larger populations are needed to confirm our results; these data suggest comparable efficacy of biosimilar FSH in the treatment of male infertility.

## Figures and Tables

**Table 1:** Characteristics of treated (cases) and untreated patients (controls) observed at the start of the study (T0) and after 3 months (T1). * In the latter column the effect of interaction time-by-group (T0–T1) is reported. § for DNA fragmentation TUNEL test: controls *N* = 50; cases *N* = 65.

Patient Characteristics	Controls (*N* = 50)			Cases (*N* = 97)			* Time Group
	T0	T1	*p*	T0	T1	*p*	*p*
Semen volume (mL)	2.9 ± 1.1	2.6 ± 1.2	0.105	2.7 ± 1.4	2.8 ± 1.3	0.054	0.008
Sperm concentration (10^6^/mL)	11.1 ± 6.7	13.4 ± 5.5	0.058	9.1 ± 10.2	12.9 ± 9.2	<0.001	0.711
Sperm total count (10^6^)	24.2 ± 16.5	26.9 ± 14.9	0.276	23.4 ± 19.8	34.4 ± 29.8	<0.001	0.013
Progressive motility (%)	14.9 ± 6.0	15.4 ± 5.2	0.482	12.6 ± 8.5	19.4 ± 12.0	<0.001	<0.001
Non-progressive motility (%)	10.0 ± 4.6	10.5 ± 4.1	0.237	9.0 ± 6.0	12.4 ± 6.9	<0.001	0.003
Immotile (%)	75.5 ± 7.6	74.8 ± 6.5	0.270	76.8 ± 15.3	51.0 ± 31.3	<0.001	<0.001
Total motile sperm count (10^6^)	7.2 ± 4.9	8.2 ± 4.2	0.230	5.5 ± 6.7	11.5 ± 13.6	<0.001	<0.001
Normal morphology (%)	6.0 ± 3.8	6.1 ± 3.2	0.578	5.8 ± 3.9	8.2 ± 4.2	<0.001	0.048
Anti-sperm antibodies (%)	4.9 ± 16.0	4.8 ± 15.2	0.695	3.1 ± 11.8	2.7 ± 10.3	0.368	0.394
FSH (U/L)	5.6 ± 1.8	5.5 ± 1.5	0.772	4.9 ± 1.9	8.2 ± 6.7	<0.001	0.032
LH (U/L)	5.3 ± 1.7	5.2 ± 1.6	0.050	4.4 ± 1.8	4.4 ± 1.7	0.860	0.385
Testosterone (nmol/L)	14.4 ± 2.7	14.5 ± 2.8	0.432	15.4 ± 4.1	15.4 ± 4.0	0.694	0.852
Right testicular volume (cc)	13.8 ± 2.7	13.8 ± 2.6	0.083	14.5 ± 5.3	15.1 ± 5.4	<0.001	<0.001
Left testicular volume (cc)	12.2 ± 2.6	12.3 ± 2.6	0.051	14.3 ± 5.4	14.6 ± 5.6	0.004	0.247
Total testicular volume (cc)	25.9 ± 4.2	26.1 ± 4.2	0.068	28.8 ± 10.4	29.7 ± 10.6	<0.001	0.001
DNA fragmentation, TUNEL test (%) §	25.8 ± 7.4	26.1 ± 6.9	0.188	24.2 ± 9.7	19.3 ± 8.0	<0.001	<0.001

**Table 2:** DNA fragmentation (%) observed at T0 and T1 in cases and controls divided on the basis of positive or negative pregnancy outcome. PGN = pregnancy, NoPGN = no pregnancy.

Pregnancy Outcome in Cases and Controls	DNA Fragmentation (%)					
	T0			T1		
	PGN	NoPGN	*p*	PGN	NoPGN	*p*
Treated patients, *N* = 65 (PGN = 10, NoPGN = 55)	15.6	25.8	0.002	12.6	20.6	0.003
Control patients, *N* = 50 (PGN = 2, NoPGN = 48)	15.5	26.2	0.043	15.5	26.5	0.027
